# Potentiometric Sensors for Iodide and Bromide Based on Pt(II)-Porphyrin

**DOI:** 10.3390/s18072297

**Published:** 2018-07-16

**Authors:** Dana Vlascici, Nicoleta Plesu, Gheorghe Fagadar-Cosma, Anca Lascu, Mihaela Petric, Manuela Crisan, Anca Belean, Eugenia Fagadar-Cosma

**Affiliations:** 1 Faculty of Chemistry, Biology, Geography, West University of Timisoara, 4 V. Parvan Ave, Timisoara 300223, Romania; danavlascici@yahoo.com; 2 Institute of Chemistry Timişoara of Romanian Academy, 24 M. Viteazul Ave, Timisoara 300223, Romania; plesu_nicole@yahoo.com (N.P.); ancalascu@yahoo.com (A.L.); mihaelapetric@yahoo.com (M.P.); mdorosencu@yahoo.com (M.C.); anca_palade@yahoo.com (A.B.); 3 Faculty of Industrial Chemistry and Environmental Engineering, Politehnica University Timisoara, Pta Victoriei 2, Timisoara 300006, Romania; gfagadar@yahoo.com

**Keywords:** Pt-metalloporphyrin, ion-selective electrode, potentiometry, iodide, bromide, PVC membrane

## Abstract

Pt(II) 5,10,15,20-tetra(4-methoxy-phenyl)-porphyrin (PtTMeOPP) was used in the construction of new ion-selective sensors. The potentiometric response characteristics (slope and selectivity) of iodide and bromide-selective electrodes based on (PtTMeOPP) metalloporphyrin in *o*-nitrophenyloctylether (NPOE), dioctylphtalate (DOP) and dioctylsebacate (DOS) plasticized poly(vinyl chloride) membranes are compared. The best results were obtained for the membranes plasticized with DOP and NPOE. The sensors have linear responses with near-Nernstian slopes toward bromide and iodide ions and good selectivity. The membrane plasticized with NPOE was electrochemically characterized using the EIS method to determine its water absorption and the diffusion coefficient into the membrane.

## 1. Introduction

Porphyrins and their derivatives have versatile physicochemical properties and structural features affording functionalization that highly recommend them as sensitive compounds for sensors design [1]. Porphyrins can recognize both positive and negative ions and also neutral molecules [2] being used for testing of biologically active substances, environmental pollutants, heavy metal ions [3] and explosives [4,5]. As a result of interaction with an analyte, the porphyrin modifies its structure and performs a change in colour, optical, fluorescent or electrical properties [6].

Potentiometric ion selective membrane electrodes [7] based on porphyrins [8] offer many advantages amongst which are: good selectivity and sensitivity, user-friendliness, lack of toxicity. Free base porphyrins perform as neutral carriers when incorporated into polymeric membranes and can detect the metallic cations by generating sitting-a-top complexes [9].

Metalloporphyrins act by selective axial coordination and can recognize and quantify several types of anions [10] and the anion selectivity can be enhanced by changing either the central metal ion or the peripheral substitution of the macrocycle [11]. 

Reports focusing on metalloporphyrins with bivalent central metal ions as ionophores for the construction of anion-selective membrane electrodes have rarely appeared in the literature and are attracting more and more attention [12].

Amongst the metalloporphyrins, platinum derivatives are receiving interest only in the last years, being successfully used for oxygen detection [13,14], perchlorate ions determination [15] and alcohol sensing [16]. The emission intensity of platinum octaethylporphyrin (PtOEP), encapsulated in a fluorinated polyimide, decreased upon exposure to ethanol and methanol vapours. Recovery is fast upon exposure to nitrogen flow. The same Pt-metalloporphyrin, incorporated in poly(1-trimethylsilyl-1-propyne) was used for trace analysis of oxygen [17,18]. Oxygen detection can be realized by monitoring the change of the intensity or lifetime of either phosphorescence or photoexcited triplet state [19]. 

Honeycomb structured porous films characterized by a large specific surface area, based on a polyfluorinated platinum tetraphenylporphyrin grafted on poly(styrene-*co*-4-vinylpyridine), were recently reported to exhibit 200% higher sensitivity than the solid sensing film. The photo-bleaching of Pt-metalloporphyrin sensing molecules, in the field of lower O_2_ partial pressures, facilitates the identification of O_2_ with the naked eye [13]. 

Another approach for maintaining long-lived phosphorescent properties involved the introduction of phenylacetylide substituents in the para position of the phenyl moieties of Pt-tetraphenylporphyrin, further incorporated in several polymers, with the purpose of improving the luminescence lifetime-based oxygen sensitivity [20]. 

Platinum(II)-5,10,15,20-*meso*-tetrakis-(2,3,4,5,6-pentafluorophenyl)-porphyrin dissolved in poly(hexafluoropropylene) (PHFP) proved to offer both higher resolution and sensitivity for oxygen sensing than previously used electrochemical sensors [21]. 

New developments in medicine rely on efficient detection in the NIR region (~1268 nm) of singlet oxygen generation using conjugated poly-(9,9-dioctylfluorene-alt-benzothiadiazole) polymer doped with platinum octaethylporphyrin (PtOEP) [22]. 

With the aim to measure CO_2_ in the gas phase [23], a solid-state sensor film based on platinum octaethylporphyrin (PtOEP) was reported.

A nanomaterial comprising graphene oxide and Pt(II)-tetraphenylporphyrin deposited on a glassy carbon electrode was developed for the selective detection of hydrazine exhibiting a wide linear range from 13 nM to 232 μM and a detection limit of 5 nM [24]. 

Using water soluble cationic Pt-porphyrin, an ultra-sensitive phosphorescence sensor for the detection of iodide ions, with a detection limit of 1 × 10^−12^ M was reported [25]. 

The literature analysis revealed only three structures of metalloporphyrins containing Pt in its II or IV state of valence, namely: Pt(II) 5,10,15,20-tetraphenylporphyrin, Pt(II) 2,3,7,8,12,13,17,18-octaethylporphyrin and Pt(IV)-tetraphenyl-porphyrin dichloride, were applied as novel ionophores for anion-selective polymeric membrane electrodes [26] although their great potential as catalysts, electrochemical mediators, photosensitizes and increasing use in optical devices is already proven [27]. The group led by Lvova demonstrated once again without equivoque that Pt(II)-porphyrins function as a neutral anion carrier in the membranes [28] and exhibit enhanced potentiometric selectivity toward iodide ions compared to electrodes based on a typical anion-exchanger (e.g., tridodecylmethylammonium chloride—TDMACl). The limit of detection for iodide anions was 10^−6^ M. 

The purpose of the present work was to synthesize and use a symmetrical *meso*-Pt(II)-porphyrin bearing electron-donating substituents, namely Pt(II) 5,10,15,20-tetra(4-methoxy-phenyl)-porphyrin, as an ionophore in different formulations of potentiometric sensors that are able to detect iodide and bromide ions in the domain of relevant concentrations for medical tests.

Bromide and iodide accurate monitoring is required in the plasma of patients and the presence of chloride might cause interferences. The detection of bromide in the presence of chloride is made after selective oxidation of bromide to bromine, the second one being detected by fluorescein using ion chromatography. Bromide monitoring has received much attention because it is used as a hypnotic or sedative drug and therapeutic levels of 10 mM are usually expected [29]. Iodide monitoring is of major interest because uptake of iodide is vital for thyroid physiology and is also a prior condition for radiotherapy with iodine for thyroid cancer [30] in levels lower than 10 µM. Iodide can arise also from iodine containing medications especially in the case of burned patients. Besides, the couple triiodide/iodide is a key redox couple used in solar-energy-harvesting systems [31] so its quantity has to be controlled.

Different methods were used to determine levels of bromide, respective iodine ions in milk and other food products, drinking water and urine [32,33] Thus, kinetic colorimetric ceric-arsenic assay [34] gas chromatography (GC) and ion chromatography (IC) [35], high performance liquid chromatography (HPLC) [36], mass spectrometry (MS) [37], inductively coupled plasma mass spectrometry (ICP-MS) [38] were used. The GC method showed higher accuracy compared to the IC procedure and provided a detection limit of 0.4 mg/L when evaluating the levels of bromide ion in urine samples of workers exposed to methyl bromide [35]. 

In the last decades, due to the vital role of selective iodide and bromide determination in various areas, non-expensive investigations using selective membrane electrodes based on different ion carriers have been done. The most extensively studied systems for sensing carriers for iodide detection are in the range of concentrations from 1 × 10^−1^ to 1 × 10^−6^ M are metallophthalocyanines [39] that can be used over a wide pH range of 3.0 ± 8.0 [40]. Approximately the same performances with metallophthalocyanines are provided by Mn(III)-metaloporphyrins [41], Co(II) and Ni(II) complexes of cyclam derivative [42], Cu (II) salicylidene complex [43] and Schiff base complexes of Cd(II) metal ions [44]. Although not improving the detection limits, another iodine sensor system focused on free base tetraphenylporphyrin incorporated into a glass-like silicone ladder-type polymer is remarkable because it provides constant selectivity coefficients for half a year [45]. 

A detection limit of 7.4 × 10^−6^ M for iodide ions and a fast response time of 15 s was obtained when Zn(II)-5,10,15,20-tetrakis(-4-pyridyl)porphyrin was used as ionophore in a liquid/polymeric membrane electrode having 20 mol % of TDMACl as additive in the membrane [46]. 

Porphyrins exhibit rich coordination properties having the capacity to distort their planar conformation through structural modification so that the internal NH groups become properly disposed for selective anion binding [47]. The group of Mamardashvili reported that the diprotonated form of 2,8,12,18-tetramethyl-3,7,13,17-tetrabutyl-porphyrin functioned as a bromide selective receptor, forming stable complexes with bromide in acetonitrile at 25 °C. Using the UV-vis spectrometric method, the limit of detection for bromide ions was of 3 × 10^−8^ M [48].

The high affinity of halide ions in complexes of 1:1 and 2:1 ratios formed with diprotonated porphyrins was also reported [49] and the binding is taking place between I^−^ ions and N^+^H groups from diprotonated porphyrins. During the halide ions binding, the porphyrin is maintaining its monomeric structure. 

Because the selective iodide and/or bromide determinations are challenges in medical, biological, environmental and food monitoring, the purpose of this research is to develop new non-toxic Pt(II) porphyrins as stable ionophores providing a wide linear response to these anions.

To achieve our purpose, Pt(II) 5,10,15,20-tetra(4-methoxy-phenyl)-porphyrin was synthesized, characterized by UV-vis, FT-IR and ^1^H-NMR spectroscopy and was used to develop new bromide and iodide ion-selective sensors. The membrane with NPOE additive was electrochemically characterized by electrochemical impedance spectroscopy regarding its water absorption and diffusion coefficient in 4-morpholinoethanesulfonic acid (MES) buffer solution. The potentiometric response of differently formulated polymeric membranes, containing Pt(II)-5,10,15,20-tetra(4-methoxy-phenyl)-porphyrin as ionophore, was investigated for different anions.

## 2. Materials and Methods

### 2.1. Reagents

For membrane preparation, poly(vinyl)chloride (PVC) of high molecular weight, bis(2-ethylhexyl)sebacate (DOS), *o*-nitrophenyloctylether (NPOE), dioctylphtalate (DOP), tridodeocylmethylammonium chloride (TDMACl) and tetrahydrofurane (THF) were purchased from Fluka (Basel, Switzerland) and Merck (Darmstadt, Germany). All salts, acids and bases were of *purum analyticum* grade. Double distilled water was used. 

### 2.2. The Synthesis and Characterization of the Ionophore Pt(II)-5,10,15,20-Tetra(4-methoxy-phenyl)-porphyrin

**Scheme 1:**
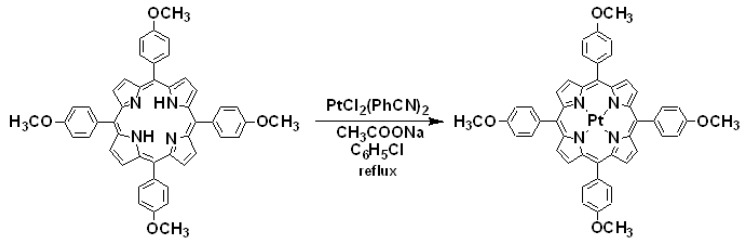
The metalation reaction of 5,10,15,20-tetra-(*p*-methoxy-phenyl)porphyrin.

The equation of the metalation reaction is given below in Scheme 1.

The method of synthesis is described, as follows: to 0.1785 g (2.429 × 10^−4^ mole) 5,10,15,20-tetra-(*p*-methoxy-phenyl)porphyrin (TMeOPP) dissolved in chlorobenzene (30 mL) was added a solution comprising 0.165 g (1.245 × 10^−3^ mole) CH_3_COONa × 3H_2_O [50] and 0.172 g (3.644 × 10^−4^ mole) PtCl_2_(PhCN)_2_ dissolved in 20 mL chlorobenzene. The reaction mixture was heated to reflux for one hour under atmospheric conditions. The metallation was monitored by UV-vis spectroscopy, until the four Q bands of the porphyrin base were reduced to only two. The reaction mixture was then cooled to room temperature and filtered. The precipitate was repeatedly washed with hot water, then collected and dried in vacuum drying oven at 90 °C for 30 h. The recrystallization was performed using CH_2_Cl_2_. Brownish-orange crystals of Pt-TMeOPP were obtained.

**Figure 1:**
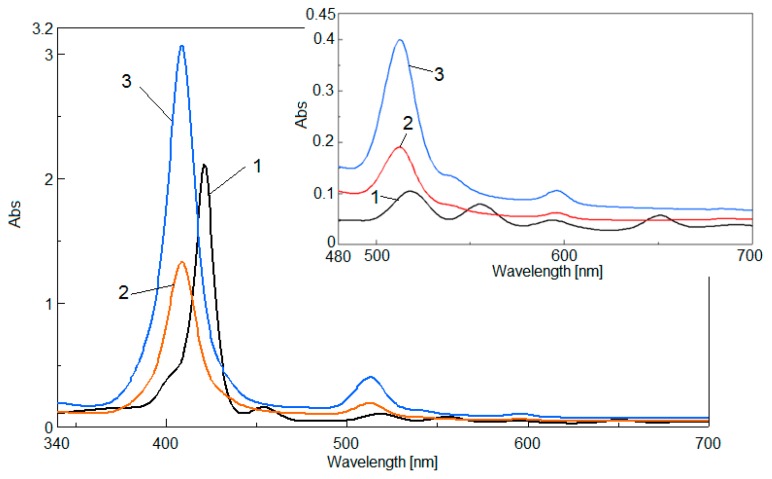
UV-vis monitoring of the metalation reaction in chlorobenzene: 1—porphyrin base; 2—Pt-metalloporphyrin generation after 15 min reflux; 3—Pt-metalloporphyrin at the end of the reaction. The Q bands in details.

As can be seen from Figure 1, the Soret band of the Pt-metalloporphyrin is significantly hypsochromically shifted and has hyperchromic effect in comparison with porphyrin-base. The same phenomena are to be seen regarding the Q bands.

The main characteristics of the (PtTMeOPP) are: brownish-orange crystals; yield 91%; mp over 320 °C; ^1^H-NMR (CDCl_3_, 500 MHz), d, ppm: 4.08 (d, 12H, 4-OCH_3_), 7.53–7.56 (m, 4H, *m*-Ph), 7.79–7.84 (m, 4H, *m*-Ph), 8.04–8.06–8.03 (dd, 8H, *o*-Ph), 8.77(d, 8H, β-Pyr); FT-IR KBr, cm^−1^: 676 (i, δpyr deform), 754 (i, δC-H *p*-substituted Ph), 1013 (i, νC-N), 1166 (i, C-O-C_asym streching_), 1242 (i, νC-O-Car), 1593 (m, νC=C); UV-vis, CH_2_Cl_2_(λ_max_ (log ε)): 409 (5.48), 513 (4.59), 596 (4.02).

From the shape and position of the signals in ^1^H-NMR it can be stated that the Pt-porphyrin is distorted, most probably in saddle type conformation and has no longer a planar structure. 

### 2.3. Sample Preparations

The performance of each sensor was investigated by measuring its potential in the concentration range 10^−6^–10^−1^ M of different anionic solutions. Stock solutions of 0.1 M were prepared by dissolving sodium or potassium salts in 4-morpholinoethanesulfonic acid (MES) of pH = 5.5. All working solutions were prepared by gradual dilution of the stock solutions.

### 2.4. Electrode Membrane Preparation and Measurements

The membranes have the composition 1% ionophore, 33% PVC and 66% plasticizer. Tridodeocylmethylammonium chloride (TDMACl) was used as additive (20 mol % relative to ionophore). The electroactive material and the solvent mediator were mixed together and then the PVC and the appropriate amount of THF were added. The ingredients were intensively stirred for about 20 min until all of them were dissolved, obtaining a transparent solution. This solution was transferred onto a glass plate of 20 cm^2^ and the THF was allowed to evaporate at room temperature leaving a tough, flexible membrane embedded in a PVC matrix. An 8 mm diameter piece of membrane was cut out and assembled on the Fluka electrode body. 

### 2.5. Apparatus and Electrodes

The measurements were carried out at room temperature using a Hanna Instruments HI223 pH/mV-meter by setting up the following cell:

Hg|Hg_2_Cl_2_|bridge electrolyte|sample|ion-selective membrane|0.01 M KCl|AgCl, Ag

Prior to EMF measurements, all the sensors were conditioned for 48 h by soaking in 0.01 M I^−^ solution. Potentiometric selectivity coefficients were determined according to the separate solution method [51] by using the experimental EMF values obtained for 0.01 M solutions of the tested anions and a theoretical slope of 59.2 mV/decade of activity. The detection limit of each sensor was established at the point of intersection of the extrapolated linear mid-range and final low concentration level segments of the calibration plot.

Electrochemical Impedance Spectroscopy (EIS) investigations were performed with the help of Autolab 302N EcoChemie equipped with the FRA2 impedance module. The sinusoidal potential amplitude was 10 mV and the tested frequency range was from 0.1 Hz to 100 kHz. All electrochemical measurements were performed at room temperature in a conventional one-compartment three-electrode cell, equipped with two stainless steel counter electrodes and Ag/AgCl as reference electrode. The working electrode is represented by the membrane fixed to a Fe electrode (active surface equal to 0.785 cm^2^). In order to determine the absorption of water, the membrane was immersed in a buffer of 2-(N*-*morpholino)ethanesulfonic acid (MES) solution. During immersion EIS spectra are recorded at OCP potential.

## 3. Results and Discussion

### 3.1. Response Characteristics of the Electrodes

In the case of platinum porphyrins, the neutral response is expected due to the 2+ oxidation state of the central ion. This is the reason why membranes with cationic additive were prepared and their responses to a number of eight anions were evaluated. The influence of three different plasticizers on the potentiometric response was also tested. The composition of the prepared membranes is presented in Table 1. Each membrane contains 20 mol % TDMACl relative to the ionophore.

**Figure 2:**
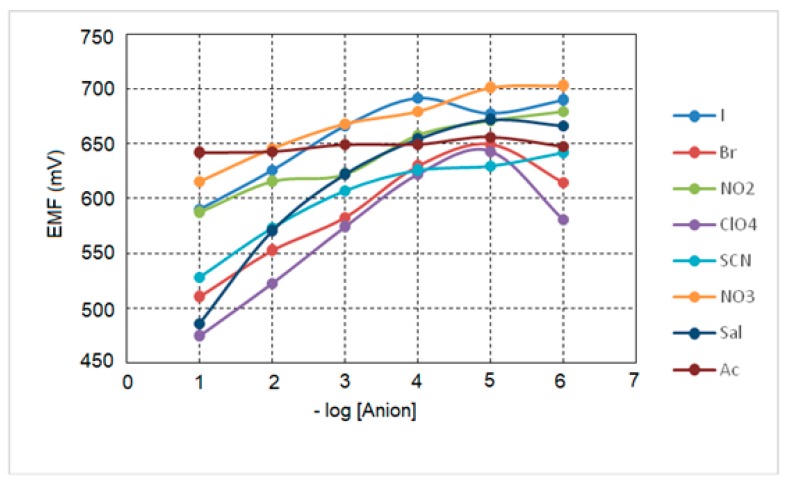
Potentiometric response of sensor A to different anions.

**Figure 3:**
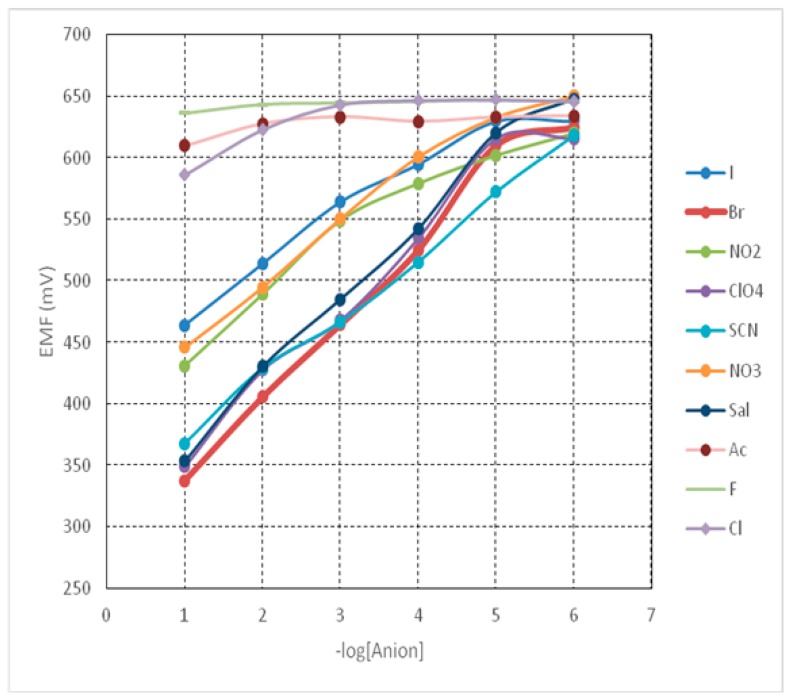
Potentiometric response of sensor B to different anions.

**Figure 4:**
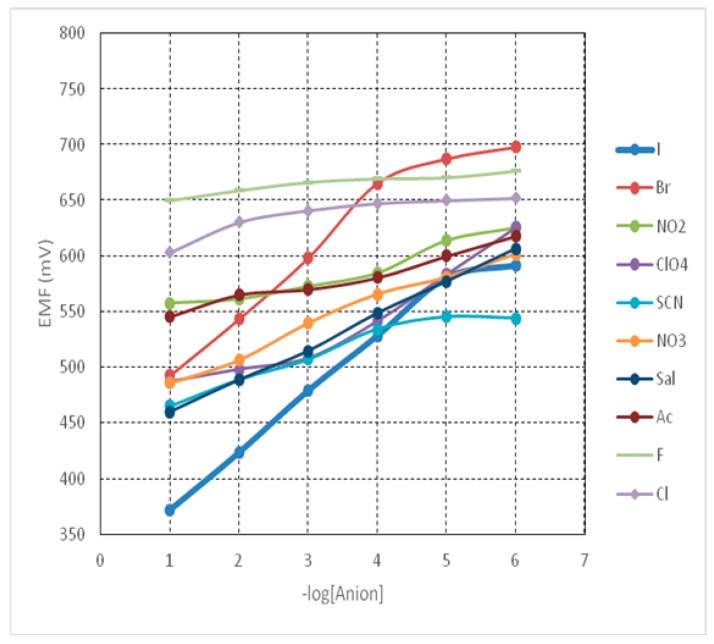
Potentiometric response of sensor C to different anions.

The potentiometric response of the sensors is presented in Figure 2, Figure 3 and Figure 4.

Analysing Figure 2, Figure 3 and Figure 4, it can be seen that the plasticizer plays an important role in the formulation of membranes. In the case of sensor A, plasticized with DOS, there is no significant potentiometric answer to any of the tested anions. It was observed that in the case of membranes containing plasticizers having a low dielectric constant such as DOS (ε = 4), the aggregation of hydrophobic porphyrins occurs and the free ability of ionophores and their complexes to move is thus constrained. In these DOS plasticized membranes the ionophores capacity to form anion-ligand complexes is diminished, conducting to randomly potentiometric response for all the tested anions (Figure 2). As a consequence, DOS plasticizer is not recommended in formulations of membranes based on this platinum (II) porphyrin destined to anions detection. 

**Figure 5:**
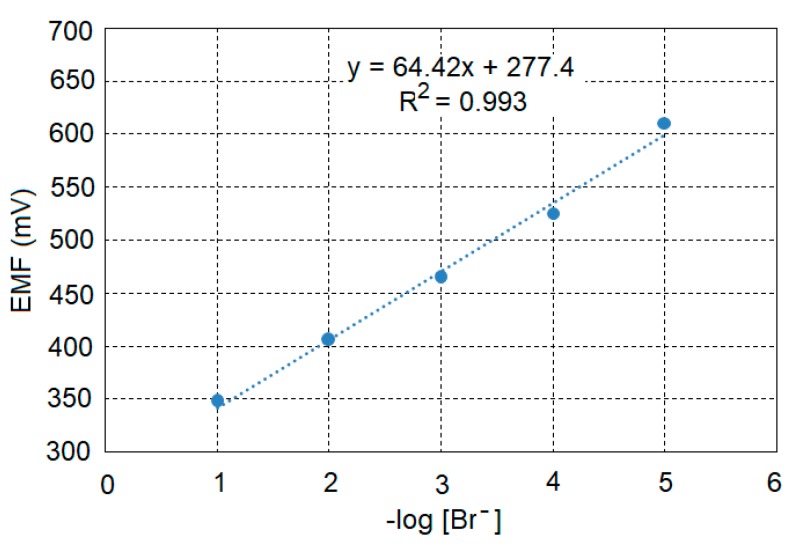
The potentiometric response of sensor B toward bromide.

The sensor B, plasticized with DOP, shows a near-Nernstian potentiometric answer to bromide in the range of 10^−1^ to 10^−5^ M with a slope of (64.4 ± 0.4) mV/decade, that is presented in Figure 5.

**Figure 6:**
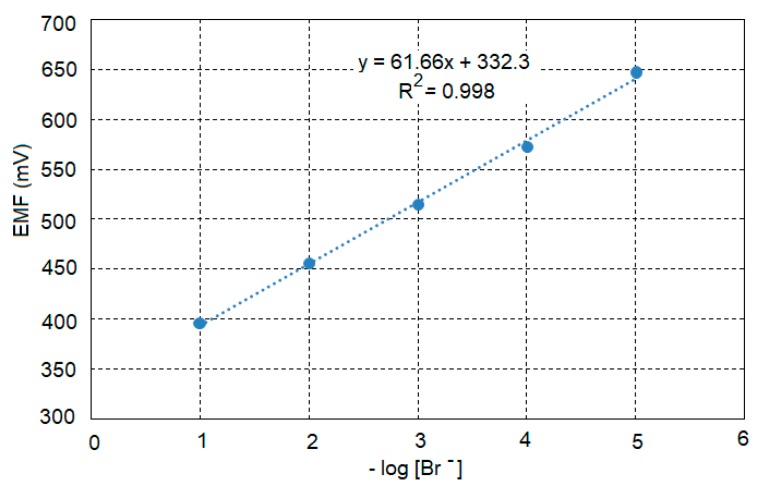
The potentiometric response of sensor B toward bromide after 4 weeks.

The sensor was intensively used for 4 weeks and after this period the slope was measured again and it is presented in Figure 6. There was a little decrease of the slope in time, (61.6 ± 0.3) mV/decade but it still remains in the analytical useful range. 

**Figure 7:**
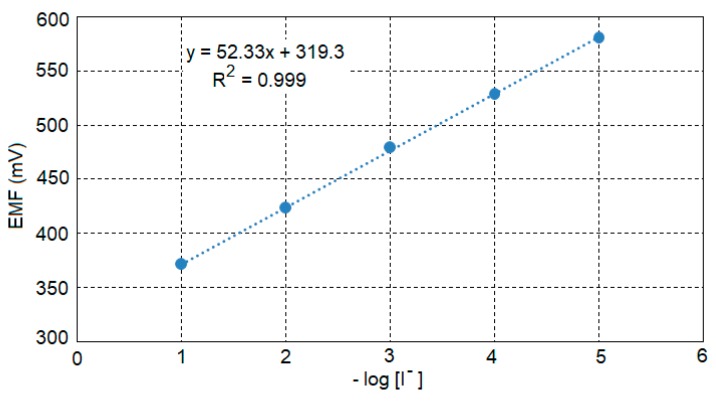
The potentiometric response of sensor C toward iodide.

In the case of sensor C, plasticized with NPOE, a potentiometric answer to iodide was obtained in the range 10^−1^ to 10^−5^ M with a near-Nernstian slope of (52.3 ± 0.2) mV/iodide decade and it is presented in Figure 7.

The sensor was also used for a period of four weeks with no significant modification of the slope value.

The nature of the plasticizer influences the dielectric constant of the membrane (*o*-nitrophenyloctylether (NPOE, ε = 24), dioctyl phtalate (DOP, ε = 7) dioctyl sebacate (DOS, ε = 4)) and the mobility of the ionophore and its complex. The porphyrins aggregation in membrane is most likely induced by the nature of plasticizer (low polar DOP and high polar NPOE plasticizer). DOP—a plasticizer with lower polarity—seems to favour the aggregation of porphyrins macrocycles and this behaviour explains the value of the Nernstian slope (the deviations from the Nernstian slope) and the preferences for bromide, an ion with a lower radius than iodide. In membranes with a polar plasticizer such as NPOE, the aggregation of hydrophobic porphyrins is hindered and the mobility of ionophores and of their complexes is increased, so that they have the capacity to form larger ion-ligand complexes, binding in this way anions with larger radius. That is the reason why this membrane gives a Nernstian response for iodide [26,52].

### 3.2. Potentiometric Selectivity

One of the most important characteristics of a sensor is the selectivity coefficient, showing the potentiometric answer of the sensor to the interfering anions toward the primary one. The selectivity coefficients of the sensors B and C, calculated using the separate solution method is presented in Table 2.

Analysing the obtained results, it can be concluded that both sensors present good selectivity compared to the interfering tested anions. 

### 3.3. Effect of the pH and the Response Time of the Sensors

**Figure 8:**
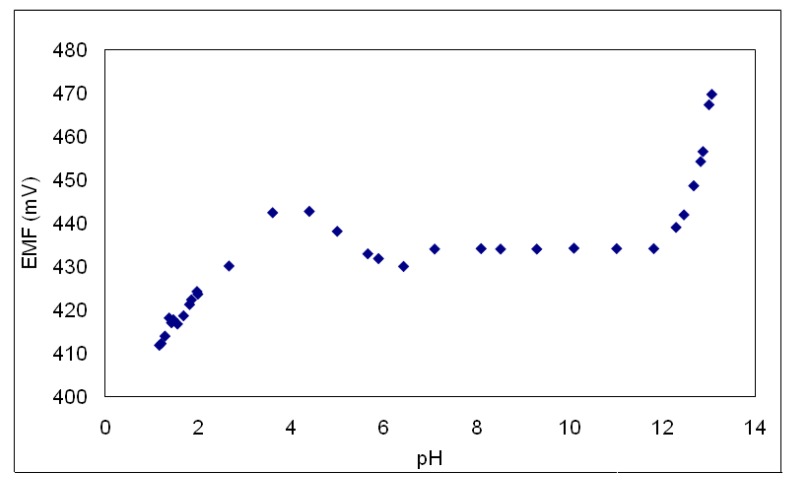
The pH function of bromide-selective sensor.

**Figure 9:**
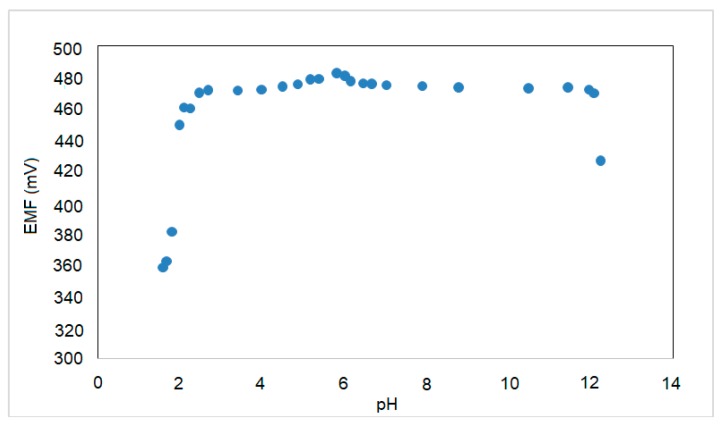
The pH function of iodide-selective sensor.

The pH functions of the two sensors were obtained by using NaOH and HCl 2 M solutions. These were added in drops to the 10^−2^ M solution of each primary anion and the behaviour is presented in Figure 8 and Figure 9.

As displayed in Figure 8, the pH range of the bromide-selective sensor is from 6–12. Figure 9 shows that the iodide-selective sensor works properly in a pH range from 3 to 12.

**Figure 10:**
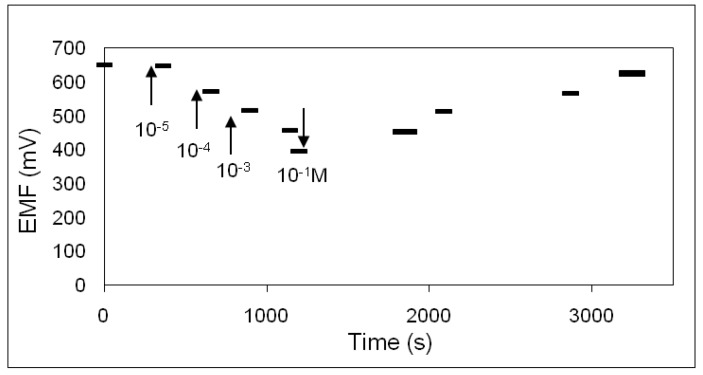
Dynamic potentiometric response to bromide and recovery time.

**Figure 11:**
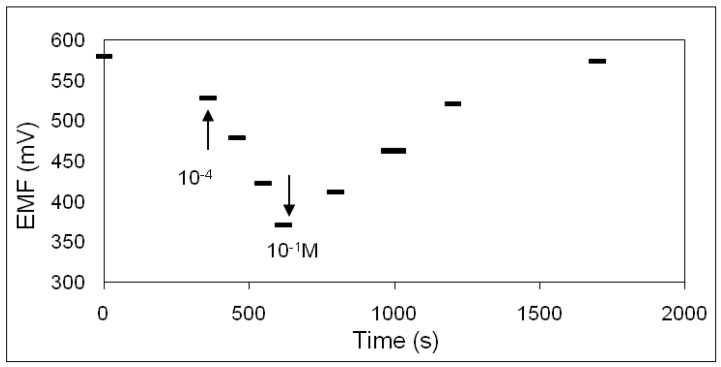
Dynamic potentiometric response to iodide and recovery time.

The average time of both sensors to reach the final potential value after successive immersions in iodide and bromide solutions, each having a 10-fold difference in concentration from low to high and reverse was measured. The results are presented in Figure 10 and Figure 11. The average times for both sensors to reach 95% of the final potential value [53] were obtained for solutions from 10^−3^ to 10^−2^ M and were of 80 s for the bromide-selective sensor, respectively 60 s for the iodide one. 

The detection limit of the electrodes was established at the point of intersection of the extrapolated linear mid-range and final low concentration level segments of the calibration plot and it is 9 × 10^−6^ M for the iodide-selective sensor and 8 × 10^−6^ M for the bromide-selective sensor. This range has relevance for the medical detection [28,29]. 

### 3.4. EIS Characterization of NPOE Membrane

In order to determine the absorption of water, the NPOE membrane was maintained in a MES buffer solution and EIS spectra were recorded during immersion at OCP, at 24 °C. The decrease of coating impedance during the immersion is expected because water and ions can penetrate into the pores of the membrane. When water penetrates the membrane, its capacitance increases as the dielectric constant increases. As a result, the capacitance can be used to measure the water absorbed by the membrane. 

**Figure 12:**
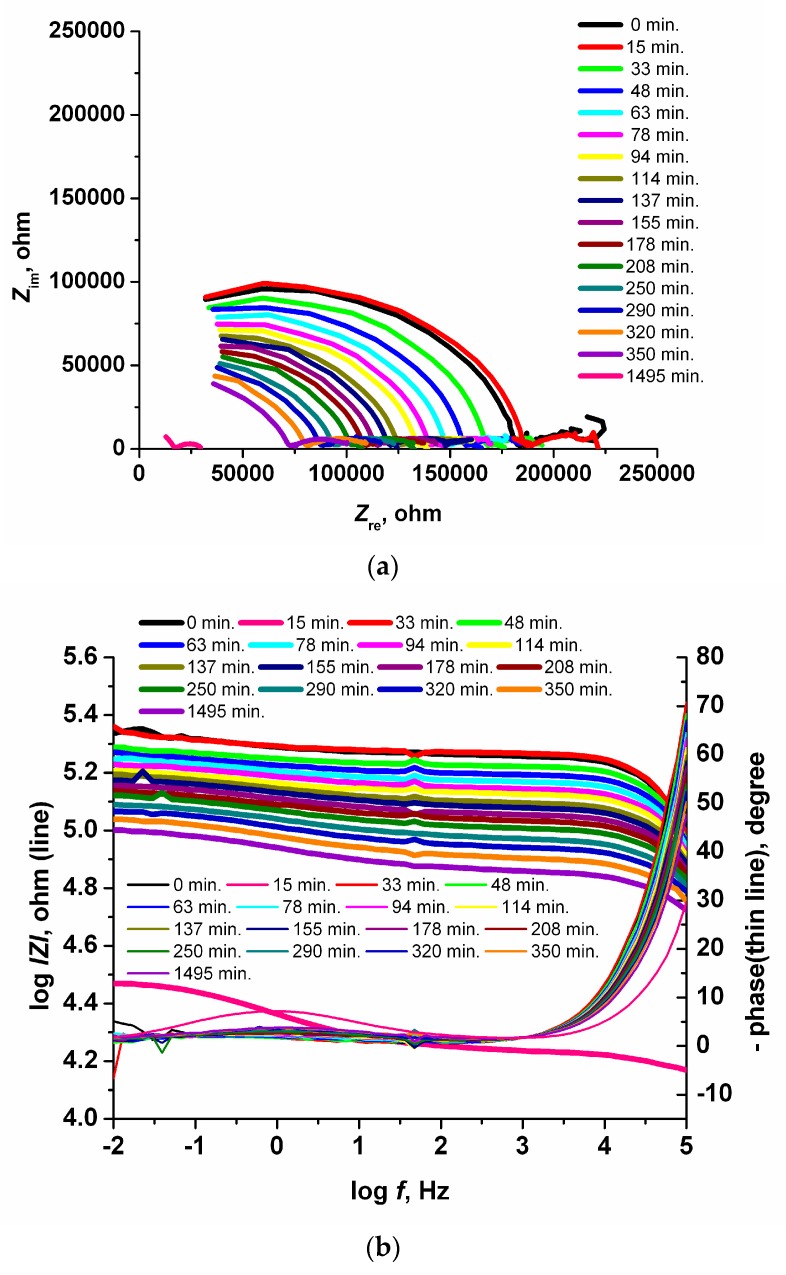
Nyquist (**a**) and Bode (**b**) diagrams in case of NPOE membrane at OCP in MES buffer solution.

The Nyquist and Bode diagrams are presented in Figure 12a,b. The immersion time for each curve is presented in Table 3. 

**Figure 13:**
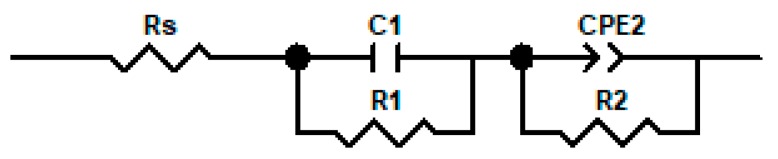
Equivalent electric circuit.

Figure 12b shows the modulus and the phase angle for the membrane during immersion. The membrane exhibited a well-defined time constant at lower frequencies (the peak in the phase angle plot). Increasing the immersion time the peak seems to move to lower frequencies. Membrane also exhibits a second peak at higher frequencies but this peak extends over the range of measured frequencies (10^5^ Hz). The experimental data were fitted to the equivalent electrical circuit by a complex non-linear least squares method, using the ZView-Scribner Associates Inc. (Southern Pines, NC, USA and Solartron Analytical, Oak Ridge, TN, USA) software. The equivalent electric circuit (EEC) used for the NPOE electrode is presented in Figure 13. The obtained results are presented in Table 3 and were used for water absorption investigation. 

The EEC includes the resistance of electrolyte R_s_, in series with a parallel connexion of capacitance C_1_ (membrane capacitance) and the resistance R_1_ (membrane resistance, similar with ionic transfer in the membrane pores) and also in series with a parallel connexion of CPE_2_ (representing the charge/discharge process that occurs at the substrate/electrolyte interface) and the polarization resistance R_2_. Constant phase element (CPE) was introduced to represent the double layer capacitance, for the reason of non-ideal behaviour. The impedance of a CPE can be expressed by Z_CPE_ = [T(jω)^n^]^−1^, where ω is frequency, T is the CPE magnitude and the exponent n is between 0 and 1. The obtained values of the electric circuit elements for the NPOE electrode are presented in Table 3.

The measurement of the water absorption or water permeability using EIS techniques is based on the determination of the changes of the coating capacitance. The absorbed water, W (volume fraction), was obtained from Equation (2) [51,54] using the membrane capacitance resulted from EIS modelling (C_t_ at different time, t, C_0_ for t = 0) and the dielectric constant of water ε_H2O_ = 80, typically at room temperature. 

(2) $$W=\frac{\log\left[C_{t}{/C}_{0}\right]}{\log80}$$

**Figure 14:**
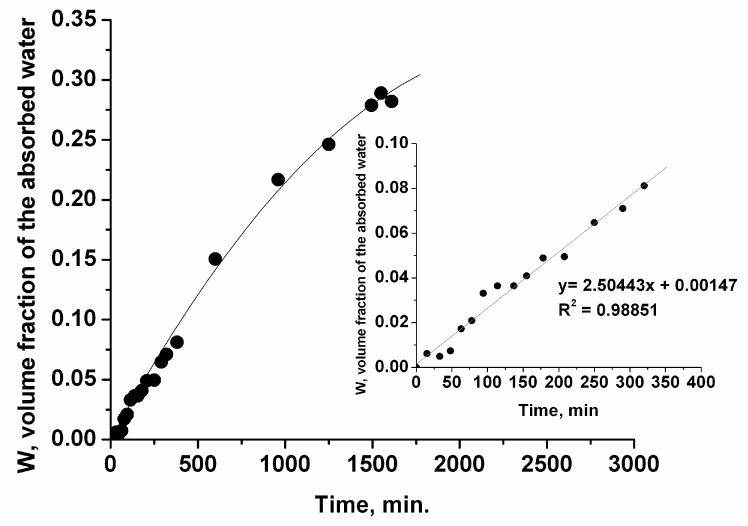
Dependence of the membrane water absorption on the immersion time. The detail presents the increase of W in the first 320 min of immersion.

The calculated data indicate an increase of the water absorption during time for the tested membrane. The water absorption (W) increase is linear until 320 min, as can be seen in Figure 14. Saturation is considered to be reached after 1495 min (~25 h). The maximum volume fraction of the absorbed water determined was 0.290.

The electrical resistance of the membrane is also influenced by the time of immersion in MES buffer solution, as the electrolyte solution penetrates through the membrane pores. It linearly decreases with the time in the first 200 min of immersion in the electrolyte solution. With the increase of immersion time the membrane resistance reaches a plateau, attributed to the saturation with water.

**Figure 15:**
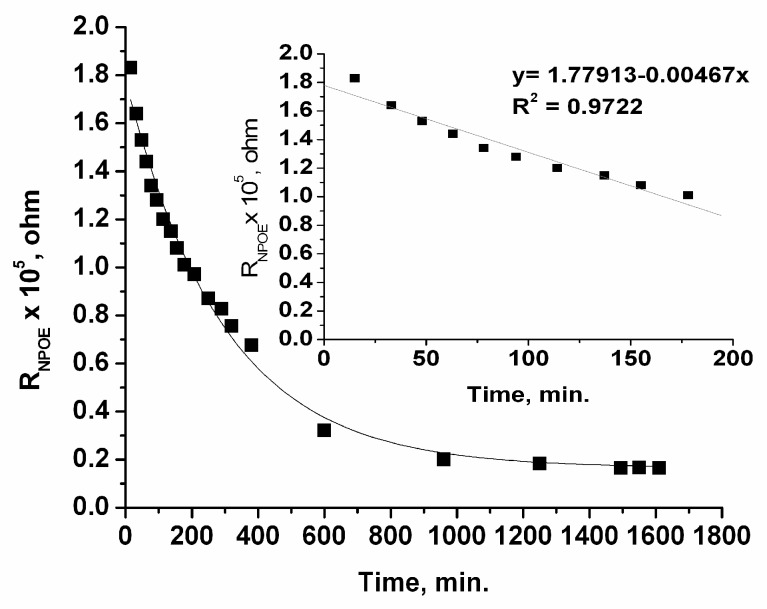
Dependence of membrane electric resistance on the immersion time in MES buffer solution. The detail presents the decrease of the membrane resistance with the time in the first 200 min of immersion.

This dependence, presented in Figure 15, is caused by the penetration of water into the membrane. The presence of water into the membrane can determine the diffusion of the electrolytes from the buffer solution into the membrane, causing also the decrease of the membrane electric resistance.

**Figure 16:**
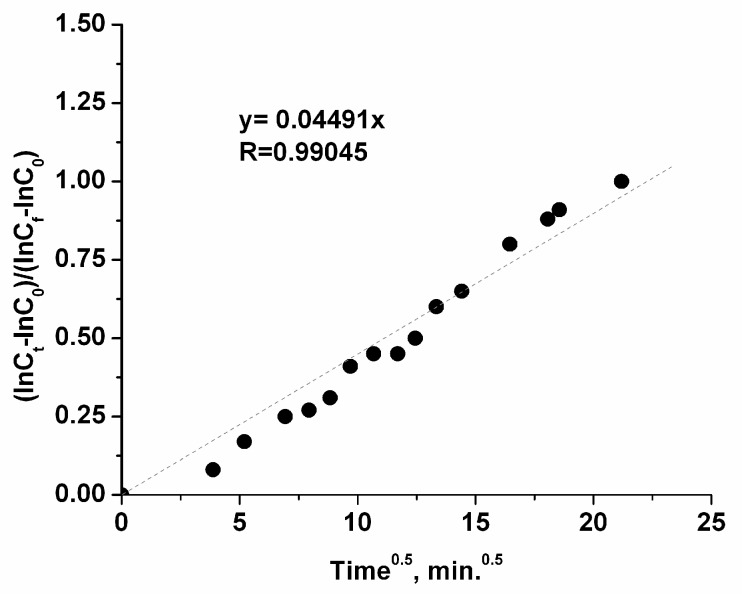
Dependence of the capacitance function $\frac{{(\mathrm{lnC}}_{t}-{\mathrm{lnC}}_{0})}{\left({\mathrm{lnC}}_{f}-{\mathrm{lnC}}_{0}\right)}$ on t^1/2^.

The diffusion coefficient, D, into the membrane was calculated according to Equation (3) [55] from the time dependence of the membrane capacitance (presented in Figure 16). C_t_ is the capacitance at the moment t, C_0_ at t = 0 and C_f_ at the final moment and L the membrane thickness. For the NPOE membrane having 180 µm, the diffusion coefficient is D = 5.13 × 10^−7^ cm^2^ s^−1^.

(3) $$\frac{{(\mathrm{lnC}}_{t}-{\mathrm{lnC}}_{0})}{\left({\mathrm{lnC}}_{f}-{\mathrm{lnC}}_{0}\right)}=\frac{2\sqrt{D}}{L\sqrt{\pi }}\sqrt{t}$$

The prepared membranes with DOP and DOS additives absorb water in a volumetric content almost identical with the NPOE membrane (the differences are no more than 0.035% comparative to NPOE).

The performed EIS measurements emphasized the necessity to introduce a membrane conditioning step, before the detection step and to establish a minimum immersion time of 25 h in MES solution.

### 3.5. Analytical Applications

Both of the sensors were used with good results for the detection of bromide and iodide from synthetic and pharmaceutical samples and the obtained values are presented in Table 4.

Additionally, the iodide-selective sensor was used for the determination of iodide in pharmaceutical potassium iodide tablets. The samples were prepared as previously described and the iodide assay was carried out by direct potentiometry. The results are presented in Table 5 (average of three measurements). The results are in good agreement with the declared label amount. 

## 4. Conclusions

There is an increased interest for the determination of iodide and bromide in therapeutic monitoring. Therefore, the objective of this study was to develop new iodide and bromide ion-selective membrane sensors. 

Presenting a very good versatility, Pt(II) 5,10,15,20-tetra(4-methoxy-phenyl)-porphyrin generates two different sensors by using different plasticizers, namely: NPOE and DOP. The calibration curves were linear in the range 10^−5^ to 10^−1^ M. The detection limit is 9 × 10^−6^ M for the iodide-selective sensor and 8 × 10^−6^ M for the bromide-selective sensor.

By EIS method the water absorption of the membranes was determined in MES buffer solution. The maximum volume fraction of the adsorbed water determined was 0.290, reached after 1495 min. The electrical resistance of the membrane decreased with the increasing of the water absorption. The performed measurements emphasized the necessity to introduce a membrane conditioning step, before the detection step and to establish a minimum immersion time of 25 h in MES solution.

A fast, sensitive and reliable potentiometric method for the determination of iodide and bromide, with relevance in the medical monitored concentration range, was developed and validated. New approaches are created by introducing the versatile PtTMeOPP as ionophore in ion-selective sensors formulations.

## Figures and Tables

**Table 1:** Membrane composition (% *w*/*w*) of the platinum porphyrin based sensors.

Sensor	Ionophore	Plasticizer			PVC
		DOS	DOP	NPOE	
A	1	66			33
B	1		66		33
C	1			66	33

**Table 2:** Selectivity coefficients of sensors B and C.

X^−^	Br	I	NO_2­­_	ClO_4_	SCN	NO_3_	Sal	Ac	Cl	F
**logK_Br,X_**	0.00	−1.83	−1.41	−0.30	−0.60	−1.51	−0.80	−3.75	−3.67	−4.01
**log K_I,X_**	−2.03	0.00	−2.34	−1.27	−1.11	−1.41	−1.10	−2.40	−3.50	−3.98

**Table 3:** Values of the electric circuit elements for the NPOE electrode.

Experiment	Time, min	Chi-Sqr	Sum-Sqr	R_s_, ohm·cm^−2^	C_1_, F·cm^2^	R_1_, ohm·cm^−2^	C_2_-T, F·cm^2^	C_2_-P, (ф)	R_2_, ohm·cm^−2^
0	0	0.004894	0.16152	0.018	1.69 × 10^−11^	1.78 × 10^5^	2.91 × 10^−5^	0.2697	161,000
1	15	0.004599	0.15178	0.018	1.74 × 10^−11^	1.83 × 10^5^	2.88 × 10^−5^	0.31686	59,954
2	33	0.004119	0.13592	0.011	1.73 × 10^−11^	1.64 × 10^5^	2.79 × 10^−5^	0.28851	47,912
3	48	0.004075	0.13449	0.009	1.75 × 10^−11^	1.53 × 10^5^	2.34 × 10^−5^	0.29763	46,669
4	63	0.002594	0.08561	0.021	1.82 × 10^−11^	1.44 × 10^5^	2.25 × 10^−5^	0.28722	52,919
5	78	0.002362	0.07795	0.019	1.85 × 10^−11^	1.34 × 10^5^	1.99 × 10^−5^	0.25473	59,122
6	94	0.002003	0.06609	0.014	1.95 × 10^−11^	1.28 × 10^5^	2.45 × 10^−5^	0.23866	63,503
7	114	0.002141	0.07066	0.018	1.98 × 10^−11^	1.20 × 10^5^	1.74 × 10^−5^	0.28806	51,832
8	137	0.004255	0.14040	0.018	1.98 × 10^−11^	1.15 × 10^5^	2.02 × 10^−5^	0.27591	69,521
9	155	0.002377	0.07845	0.018	2.02 × 10^−11^	1.08 × 10^5^	1.70 × 10^−5^	0.27767	51,633
10	178	0.002051	0.06769	0.003	2.09 × 10^−11^	1.01 × 10^5^	1.79 × 10^−5^	0.23961	56,215
11	208	0.002489	0.08213	0.018	2.10 × 10^−11^	97,137	1.62 × 10^−5^	0.29857	47,008
12	250	0.001946	0.06422	0.018	2.24 × 10^−11^	87,083	1.64 × 10^−5^	0.25399	54,603
13	290	0.001997	0.06590	0.018	2.31 × 10^−11^	82,704	1.67 × 10^−5^	0.29825	48,680
14	320	0.001972	0.06506	0.018	2.41 × 10^−11^	75,544	1.59 × 10^−5^	0.30225	45,276
15	380	0.001752	0.05783	0.019	2.52 × 10^−11^	67,552	1.72 × 10^−5^	0.28623	45,792
16	1495	0.001962	0.06476	0.019	6.00 × 10^−11^	16,596	3.98 × 10^−5^	0.43701	15,680

**Table 4:** Analytical applications of sensors from synthetic samples.

Synthetic Sample	Amount (mol/L)	Found by Electrode (mol/L ± S ^a^)
Iodide	7.5 × 10^−4^	(7.4 ± 0.1) × 10^−4^
Bromide	5.0 × 10^−4^	(5.2 ± 0.2) × 10^−4^

^a^ Average of determinations on three samples of the same origin.

**Table 5:** Determination of iodide in pharmaceutical formulations.

Sample	Label Amount (µg)	Found by Potentiometry (µg)
Potassium iodide tablets (Merck)	130.8	129.7 ± 0.9
